# Genetic architecture is more complex for resistance to Septoria tritici blotch than to Fusarium head blight in Central European winter wheat

**DOI:** 10.1186/s12864-015-1628-8

**Published:** 2015-06-05

**Authors:** Vilson Mirdita, Guozheng Liu, Yusheng Zhao, Thomas Miedaner, C. Friedrich H. Longin, Manje Gowda, Michael Florian Mette, Jochen C. Reif

**Affiliations:** Department of Cytogenetics and Genome Analysis, Leibniz Institute of Plant Genetics and Crop Plant Research (IPK), 06466 Gatersleben, Germany; State Plant Breeding Institute, University of Hohenheim, 70593 Stuttgart, Germany; International Maize and Wheat Improvement Center (CIMMYT), P. O. Box 1041–00621, Nairobi, Kenya

**Keywords:** Association mapping, Genomic selection, Hybrid wheat, Fusarium head blight, Septoria tritici blotch

## Abstract

**Background:**

Fusarium head blight (FHB) and Septoria tritici blotch (STB) severely impair wheat production. With the aim to further elucidate the genetic architecture underlying FHB and STB resistance, we phenotyped 1604 European wheat hybrids and their 135 parental lines for FHB and STB disease severities and determined genotypes at 17,372 single-nucleotide polymorphic loci.

**Results:**

Cross-validated association mapping revealed the absence of large effect QTL for both traits. Genomic selection showed a three times higher prediction accuracy for FHB than STB disease severity for test sets largely unrelated to the training sets.

**Conclusions:**

Our findings suggest that the genetic architecture is less complex and, hence, can be more properly tackled to perform accurate prediction for FHB than STB disease severity. Consequently, FHB disease severity is an interesting model trait to fine-tune genomic selection models exploiting beyond relatedness also knowledge of the genetic architecture.

**Electronic supplementary material:**

The online version of this article (doi:10.1186/s12864-015-1628-8) contains supplementary material, which is available to authorized users.

## Background

Wheat improvement is characterized by the need to control a high-dimensional trait space comprising grain yield and its components, quality traits, as well as abiotic and biotic stress resistances [1]. As a consequence of the large number of relevant traits, multi-stage selection is commonly applied in wheat breeding programs. Multi-stage selection encompasses classical phenotypic selection as well as marker-assisted [2] and genomic selection [3, 4].

In marker-assisted selection, a small number of predefined functional markers is used to predict the performance of genotyped material for a trait [5]. This strategy is worthwhile if major quantitative trait loci (QTL) are present that contribute to a large proportion of the genotypic variance for traits which are difficult and expensive to phenotype [2]. In contrast, in genomic selection, a large number of markers is used to predict the performance for complex traits controlled by many QTL with small effects [6, 7]. For traits with presence of a few large and several small effect QTL, alternative biometrical approaches have been proposed to maximally profit from a combined marker-assisted and genomic selection [8, 9].

Fusarium head blight (FHB) caused by *Fusarium graminearum*, *F. culmorum* and other *Fusarium* species severely impacts wheat production worldwide [10]. Septoria tritici blotch (STB) disease caused by *Mycosphaerella graminicola* (anamorph *Septoria tritici*) has become one of the most devastating leaf diseases in Central European winter wheat [11]. Accurate knowledge of the genetic architecture of FHB and STB resistance is needed for a custom-tailored design of genomics-based breeding strategies. In this context, it is important to take into account that prediction accuracy of genomic selection is not only driven by the linkage disequilibrium between molecular markers and QTL, but also exploits genetic relatedness between members of the training and the test population [12]. Especially in studies based on small training population sizes, effects of relatedness may become of overwhelming importance [12, 13]. Consequently, genomic selection models are not necessarily stable across different cycles of selection.

The relative relevance of linkage disequilibrium in comparison to relatedness for genomic selection is trait specific. It is challenging to differentiate both sources contributing to the accuracy of genomic selection as their effects are intermingled [14]. Using mapping populations derived from factorial crosses offers a unique opportunity to untie linkage disequilibrium from relatedness as test populations with defined gradual degrees of relatedness to the training population can be established [15].

The genetic architecture of STB resistance has been recently analyzed based on a mapping population of approximately 1000 European wheat hybrids that were phenotyped in two environments [11]. The related cross-validation study suggested that the genetic architecture underlying STB resistance in this population is complex with absence of large effect QTL, which is in accordance to previous findings [16–18]. The failure to detect large effect race-specific resistance genes such as *Stb1* originating from a Bulgarian landrace [19] in this collection can be explained by the fact that the related favorable alleles have not yet been introgressed into European elite wheat lines [11]. The potential of genomic selection in the above mentioned hybrid population was previously found to substantially depend on genetic relatedness [11]. The accuracy to predict STB resistance amounted only to 0.3 when using a test set mostly unrelated to the training set. This accuracy is surprisingly low and comparable to that observed for grain yield in a similar experimental make-up [20]. Verification of this result is relevant in order to assess the stability of genomic selection models for STB resistance.

Major QTL for FHB resistance have been identified in populations derived from crosses with exotic donor lines. Examples comprise QTL *Fhb1* from line Sumai 3 or QTL *Fhb5* from line CM82036 [21]. Despite worldwide efforts, however, the favorable alleles of these QTL have so far not been used in wheat breeding in Central Europe [22]. As alternative strategy, identification of alternative QTL in adapted European wheat germplasm has been approached based on biparental QTL mapping [10, 23, 24] and association mapping [25–27]. The lack of congruency of QTL results across studies [24], however, points to the presence of multiple QTL exhibiting small effects. First experimental results on the potential of genomic selection suggested that precise calibration can be achieved to improve FHB resistance in wheat breeding [27, 28]. Nevertheless, it is not clear whether the obtained calibration models mainly exploited relatedness between training and test sets and to what extent functional QTL information contributed to the prediction model.

Based on phenotypic data obtained from multi-environment field trials and genotypic data generated using a wheat 90 k SNP array for a large collection of 1604 F1 elite winter wheat hybrids and their 135 parental inbred lines, we contrasted the genetic architecture of FHB and STB disease severities applying association mapping and genomic selection in combination with a cross-validation approach. The objectives of our study were to (1) examine the correlations among FHB and STB disease severities, (2) investigate the genetic architecture of both traits, and (3) assess the potential of marker-assisted and genomic selection for improving FHB and STB disease resistance.

## Results

### Extensive field evaluation resulted in high heritabilities of FHB and STB disease severities

In the three environments Harzhof 2012, Harzhof 2013 and Rosenthal 2013 FHB and STB disease severities were scored on the same plots, but we observed no significant correlations between FHB and STB disease severity values for all three environments (Additional file 1: Figure S1) and Consequently: This finding is in accordance to a previous study in wheat investigating the potential to simultaneously test for FHB and STB resistance in the same plot [29], it is unlikely that the combined evaluation of both diseases impaired the quality of phenotypic data, thus representing an efficient phenotyping strategy.

FHB and STB disease pressures were high in all environments as reflected by the wide range of phenotypic values and genotypic variances significantly (*P < 0.05*) larger than zero observed for all environments (Additional file 1: Figure S1). The Pearson moment correlations among phenotypic values of the 1604 hybrids and their 135 parental lines estimated for single environments were on average moderate (*r* = 0.31, *P* < 0.01 for FHB disease severity and *r* = 0.31, *P* < 0.01 for STB disease severity). These levels of Pearson moment correlation are characteristic for diseases where susceptibility is controlled by multiple gene loci. Consequently, multi-location field trials are needed to precisely estimate the genotypic value for FHB and STB disease severity.

**Fig. 1:**
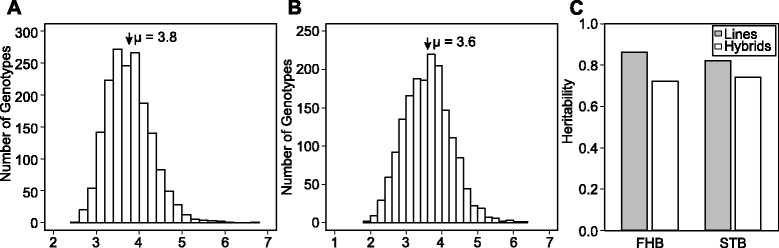
Distribution of FHB and STB disease severities. **a** Fusarium head blight (FHB) and **b** Septoria tritici blotch (STB) at a rating scale from 1 to 9 (1 = healthy plants; 9 = 100 % infected plants) as well as the estimates of broad-sense **c** heritability for the population of 1739 wheat genotypes (1604 hybrids and 135 parental lines) evaluated in up to seven environments. μ indicates average of diseases severities. Heritabilities where calculated in broad sense

**Fig. 2:**
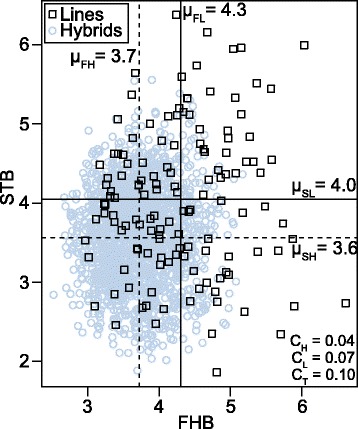
Association between Fusarium head blight (FHB) and Septoria tritici blotch (STB) disease severities. The data correspond to the best linear unbiased estimates of 1604 wheat hybrids (circle) and their 135 parental lines (squares) evaluated in up to seven environments. Lines labeled μ_FL_ and μ_FH_ indicate the average of FHB severities for parental lines and hybrids, respectively, lines labeled μ_SL_ and μ_SH_ indicate the average of STB severities. C_L_, C_H_, and C_T_ indicate correlations of both disease severities in parental lines, hybrids, and the population in total

In the analyses across environments, we observed a wide range of FHB and STB disease severity values approximating a normal distribution (Figs. 1 and 2). This suggests the absence of large effect QTL, which, if present, should be reflected by discrete phenotype classes. The genetic variances of FHB and STB disease severities were significantly (*P < 0.01*) larger than zero. Heritability estimates for lines and hybrids were high and similar for both traits (Fig. 1). This clearly underlined the excellent quality of the phenotypic data, that thus should be suitable to investigate the genetic architectures of both traits *via* association mapping and genomic selection.

### Simulation study suggests a high power to detect major QTL

**Fig. 3:**
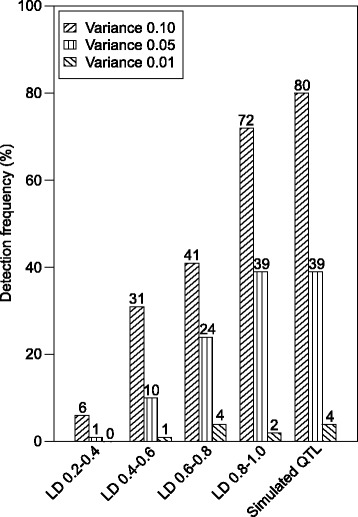
Simulations study of QTL detection power. Detection frequency of a simulated QTL explaining 1 % (0.01), 5 % (0.05), or 10 % (0.1) of the genotypic variance, assuming markers in linkage disequilibrium (LD) with the simulated QTL within r^2^ value classes 0.2–0.4, 0.4–0.6, 0.6–0.8, and 0.8–1.0

The potential power of our experimental setting to detect QTL was explored using simulations using detection frequency as a measure (Fig. 3). For small or intermediate effect QTL explaining 1 % or 5 % of the genotypic variance, detection power was predicted to be low to moderate. In contrast, for QTL explaining 10 % of the genotypic variance, detection power is predicted high provided the presence of SNPs in tight linkage disequilibrium to the QTL with *r*^*2*^ values above 0.8. The average genetic map distance among adjacent SNP pairs amounted to 0.72 cM in our study, with average *r*^*2*^ values of 0.54 (Additional file 2: Figure S2). Consequently, QTL detection power in our study was limited by the available marker density and might have been enhanced by increasing the genotyping depths of the 135 parental lines.

### Marker-assisted selection did not facilitate precise prediction of FHB and STB disease severities in unrelated genotypes

**Fig. 4:**
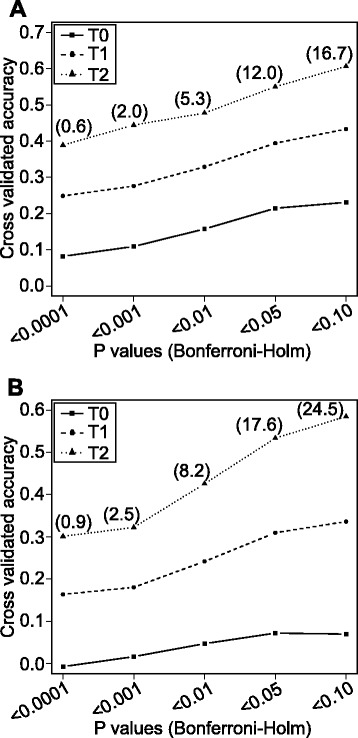
Cross-validated accuracies of prediction for marker-assisted selection of **a** Fusarium head blight and **b** Septoria tritici blotch disease severity. Markers included were selected based on different levels of significance (P values) of associations. T2 test sets included hybrids sharing both parental lines, T1 test sets hybrids sharing one parental line and T0 test sets hybrids having no parental line in common with the hybrids in the related training sets. Numbers in brackets indicate the average number of significant marker–trait associations based on 100 cross-validation runs

Cross-validated accuracies of prediction for FHB and STB disease severities obtained by association mapping largely differed depending on the relatedness of members of training and test sets and the significance thresholds applied (Fig. 4). For the T2 scenario with the test set highly related to the training set, we observed more than fourfold larger accuracies of prediction in comparison to the T0 scenario involving an unrelated test set for both traits. The cross-validated accuracies of prediction were for both traits close to zero with a stringent significance threshold for the T0 test population level and increased only slightly with relaxed significance thresholds.

### Genomic selection allowed more accurate prediction of FHB and STB disease severities than marker-assisted selection

**Fig. 5:**
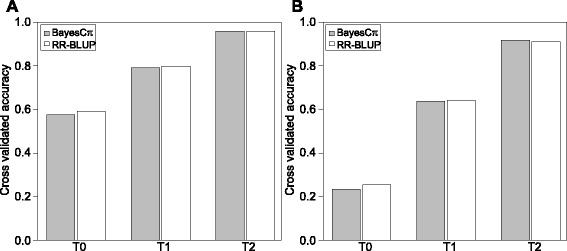
Cross-validated accuracies of prediction for genomic selection of **a** Fusarium head blight and **b** Septoria tritici blotch disease severity in wheat. The results are based on the two genomic selection models BayesCπ and ridge regression Best Linear Unbiased Prediction (RR-BLUP). T2 test sets included hybrids sharing both parental lines, T1 test sets hybrids sharing one parental line and T0 test sets hybrids having no parental line in common with the hybrids in the related training sets

Cross-validated accuracies of prediction of disease severity among unrelated hybrids (T0 scenario) were low to moderate and amounted to 0.58 for FHB and for 0.23 STB (Fig. 5). Among related hybrids T2 scenario, accuracies of prediction accuracy increased 1.6 - and 3.6 - fold, respectively for FHB and STB disease severity. Both genomic selection models applied, BayesCπ and RR-BLUP, resulted in very similar prediction accuracies. A combined prediction approach considering additive and dominance effects was only slightly superior in predicting FHB and STB disease severities than the approach based on additive effects only (Additional file 3: Table S1).

## Discussion

### Independent genetic architectures of FHB and STB disease severities

Miedaner et al. [29] suggested that in European elite wheat lines FHB and STB disease severities are only marginally influenced by pleiotrophic and linkage effects, resulting in low genotypic correlations. Our results confirm this view, as no significant correlation (*r =* 0.10; *P > 0.05*) between both disease severities was detected (Fig. 2). In consequence, multivariate approaches modeling the covariance among traits as suggested for instance by [30] cannot be expected to improve neither phenotypic analysis, nor association mapping or genomic selection approaches. Therefore, we focused on univariate analyses of the genetic architecture of FHB and STB disease severities.

### Genetic architecture of FHB is less complex than for STB disease severity in Central European elite wheat

Cross-validated association mapping results are known to be influenced by relatedness between training and test populations as well as by functional QTL information [27]. The use of a hybrid population produced based on factorial mating designs enables the disentaglement of the two factors by inspecting the accuracy of prediction in a T0 test set largely unrelated to the estimation set in comparison to more related T1 and most related T2 test sets [15]. In our study, we failed to detect major QTL for both traits, which was reflected by a low accuracy of prediction of marker-assisted selection even in the T0 scenario (Fig. 4). This clearly suggests that even though several major QTL controlling FHB and STB disease severities have been identified in exotic genotypes [10, 19, 21, 31], none of them is currently exploited in the sampled European elite varieties. The absence of large effect QTL from exotic donors in the Central European wheat lines is most likely due to substantial yield penalties [22].

The similar accuracy of prediction of genomic selection of FHB versus STB disease severity for the T2 scenario (Fig. 5), in which relatedness between training and test sets is mainly exploited [15], is not surprising as the underlying phenotypic data were of similar precision (Fig. 1C). In contrast, the accuracy of prediction of genomic selection was nearly 3 times larger for FHB than STB disease severity for the T0 test set most unrelated to the training set (Fig. 5). The observed difference can be explained mainly by a better exploitation of functional QTL information for FHB than for STB disease severity. Thus, our findings suggest that the genetic architecture is less complex in the case of FHB and, hence, given the same level of relatedness between training and test sets, can be more properly tackled to predict FHB than STB disease severity.

### BayesCπ does not improve the accuracy of prediction for FHB disease severity, a trait with medium genetic complexity

From the two genomic selection approaches tested, RR-BLUP approximates the infinitesimal model using the same shrinkage factor for all markers [32]. In contrast, BayesCπ performs variable selection and assumes that a fraction 1-π of markers is not contributing to the genetic variance [33]. Considering the lower complexity of the genetic architecture of FHB than STB disease severity reflected by three times higher prediction accuracies in the unrelated T0 test sets (Fig. 5), it could be expected that BayesCπ would outperform RR-BLUP. This was, however, not the case in our study. A possible explanation would be that several SNPs in linkage disequilibrium to relevant QTL were counterbalancing the drawbacks of RR-BLUP [34]. Moreover, the precision of estimating the hyper parameters π tends to be overestimated [33], which could reduce the advantages of BayesCπ in comparison to RR-BLUP in modelling the genetic architecture of FHB disease severity more properly.

## Conclusions

The diseases FHB and STB severely impair wheat production worldwide. Both traits require intensive field trials to precisely estimate genotypic values, making them interesting targets for genomics-assisted breeding. Our results suggest that the genetic architectures of both traits are complex, which favors genomic versus marker-assisted selection.

Contrasting the cross-validated accuracies of prediction observed for tests sets with different degree of relatedness to the training sets clearly underlines that the precision to predict STB disease severity is mainly driven by relatedness. In contrast, genomic selection models are still moderately accurate predicting FHB disease severity in largely unrelated populations. Consequently, functional QTL variation is exploited, which makes FHB disease severity an interesting model trait to fine-tune genomic selection models tackling besides relatedness also knowledge of the genetic architecture. One promising option to improve the prediction accuracy for FHB disease severity for instance, consists in expanding the Bayesian alphabet by specifying distinct prior distributions for small and large marker effects.

## Methods

### Plant materials and field trials

Fifteen European winter wheat (*Triticum aestivum* L.) lines were crossed as males in a factorial scheme with 120 female lines by the use of chemical hybridization agents, yielding sufficient seed for field trials from 1604 F_1_ hybrids [35]. The genetic make-up of this population of parental lines and hybrids has been described in detail with regard to a variety of traits [8, 11, 15, 20, 36].

The 135 parental lines and 1604 hybrids derived from them were evaluated in parallel with 10 released reference varieties for FHB disease severity in five environments (location × year combinations, Additional file 1: Figure S1). A reduced set comprising the 135 parental lines, 1055 hybrids and the 10 released reference varieties was evaluated in further two environments. Test designs were unreplicated in 5 environments, partially replicated in one environment, and replicated in one environment. In all environments, lines and hybrids were artificially spray inoculated except in Harzhof 2012, where scoring was based on natural infection. FHB inoculum production based on autoclaved wheat kernels as substrate was performed as described in detail by [37]. Directly before inoculation, spores were rinsed off with tap water, counted, and diluted for spray inoculation with a common plot sprayer. To compensate for different heading times of the involved wheat genotypes, inoculation was carried out in total three to four times in intervals of three to four days, starting when the first 20 % of genotypes were flowering. Thus, inoculation of each genotype at least once at full flowering was facilitated. The inoculum had a concentration of 5 × 10^4^ spores mL^−1^ for Rosenthal 2012 and 2013 and of 2 × 10^5^ spores mL^−1^ for all other location-year combinations. FHB infection was scored in an ordinal scale of 1 to 9, where 1 refers to healthy plants and 9 stands for 100 % infected plants. In Böhnshausen 2012, Böhnshausen 2013, and Hohenheim 2013, FHB infection was scored at different time intervals, with the mean of these scorings used for analysis. In the other environments, disease severity data were recorded at a single date with optimal differentiation among the entries.

The population of 1749 genotypes was also evaluated for STB disease severity at three locations in the year 2012 and at five locations in the year 2013 (Additional file 1: Figure S1). Test locations were Hadmersleben, Harzhof, Rosenthal, Seligenstadt and Cecilienkoog. In Cecilienkoog, only a reduced set of 1200 genotypes (1055 hybrids, 135 parental lines, and 10 released reference varieties) was evaluated for STB disease severity. The experimental design was an alpha design where replication and location effects were confounded. In Cecilienkoog, targeted inoculation with a mixture of isolates was performed by spraying a spore suspension with a concentration of 1 × 10^6^ spores mL^−1^ for one time when all genotypes had fully expanded flag leaves. In Rosenthal, Hadmersleben, and Harzhof, natural STB infection facilitated by cultivation of susceptible spreader varieties was followed. STB disease severity was visually scored plot wise as coverage of flag leaves with lesions bearing pycnidia on a scale from 1 (fully resistant) to 9 (fully susceptible).

### Phenotypic data analyses

Evaluations for FHB and STB disease severities were performed in up to seven environments reflecting a combination of replicated and unreplicated trials owing to the high number of entries coupled with limited phenotyping capacities. Therefore, in the analysis of variance, we estimated best linear unbiased estimates (BLUEs) separately for the two environments with replicated data. In the next step, we used these BLUEs along with the raw data from the other five environments without replications and carried out an analysis of variance across environments as outlined in detail in [38]. In addition, we assumed fixed genotypic effects to obtain BLUEs of the genotypic values. All statistical analyses were performed by the restricted maximum likelihood method using ASReml version 3.0 [39].

### Genotypic data

DNA was extracted according to standard procedures from all genotypes and fingerprinting was performed with a 90 k SNP array based on an Illumina Infinium assay [40]. All markers that were either monomorphic, had missing values of >5 %, heterozygosity of >5 % in inbred lines, or had a minor allele frequency of <5 % were discarded from analysis [20]. After this filtering, 17,372 high-quality SNP markers were retained (Dryad Digital Repository: doi:10.5061/dryad.461nc).

### Genome-wide mapping

Data from each environment were used in association mapping scans correcting for population stratification with a kinship matrix [20]. Kinship matrices for the inbred lines and hybrids were modelled as described previously [20, 41]. Genome-wide scans for marker–trait associations were conducted to detect main-effect QTL. The Bonferroni-Holm procedure [42] was applied to correct for multiple testing at different significance levels.

Based on the adjusted entry means of all genotypes, we applied Ridge Regression Best Linear Unbiased Prediction (RR-BLUP) [43] and BayesC *π* [33, 44] considering additive and dominance effects. Details of the implementation of the models have been described in [20]. All statistical procedures for the genomic selection approaches were executed using [45].

### Cross validation

We evaluated the accuracy of prediction of FHB and STB disease severities by association mapping and two genomic selection approaches RR-BLUP and BayesC *π* using cross validations. In this work, we sampled 100 times 600 hybrids, 10 of their male, and 80 of their female parental lines as the training set, and estimated the additive and dominance effects applying the genomic selection as well as the association mapping models outlined above. For the association mapping approach, we corrected for population stratification with a kinship matrix and identified significant marker-trait associations. The implementation of the two genomic selection models was based on estimates of the genetic variances and the broad-sense heritability on an entry-mean basis of the full population. We tested the accuracies of prediction for three types of test sets with gradually decreasing degrees of relatedness to the training set. The most closely related test set T_2_ included different hybrids derived from the same parental lines as the hybrids evaluated in the training set, while the less related test set T_1_ included hybrids sharing one parental line with the hybrids in the training set and the least related test set T_0_ included only hybrids having no parental lines common with the hybrids in the training set [15]. Prediction accuracy was estimated as Pearson’s correlation coefficient between the observed and the predicted hybrid performance standardized with the square root of the broad-sense heritability on an entry-mean basis.

### Simulation study

We performed a simulation study to examine the power to detect QTL in our association mapping approach using our 90 k SNP array data. Following [27], we simulated a trait with a heritability of 0.7 and assumed the presence of one main effect QTL with an allele frequency of 0.1, explaining 1 %, 5 %, or 10 % of the genotypic variation. We performed 200 simulation runs for each scenario, conducing genome-wide scans for marker-trait associations to detect main-effect QTL applying a Bonferroni-Holm procedure correcting for multiple testing at an significance level of 0.01, and recorded the frequency of QTL detection as well as the detection of SNPs which are in linkage disequilibrium with the simulated QTL (r^2^ values classes: 0.2–0.4, 0.4–0.6, 0.6–0.8, 0.8–1.0).

### Data availability

The data is stored under Dryad Digital Repository: doi:10.5061/dryad.461nc.

### Ethics statement

This study did not involve taking actual samples from humans or animals.

## Additional files

Additional file 1: Figure S1. Correlation of FHB and STB disease severities between the environments. Heat plots of Pearson moment correlation coefficients among single environments entry-means for (A) Fusarium head blight and (B) Septoria tritici blotch disease severity of the 1749 wheat genotypes (1604 hybrids, their 135 parental lines, and 10 released reference varieties). Additional file 2: Figure S2. Marker density and linkage disequilibrium (LD). Genetic map distance between adjacent markers (A) and extent of LD among adjacent marker pairs measured (B). Additional file 3: Table S1. Average accuracies of prediction of cross-validated explained genotypic variance for BayesCπ and RR-BLUP for resistance against Fusarium head blight (FHB) and Septoria tritici blotch (STB) based on genotyping data from a 90 k SNP array.
