# Supplementary material for: Genetic architecture is more complex for resistance to Septoria tritici blotch than to Fusarium head blight in Central European winter wheat
Source: BMC Genomics. 2015 Jun 5;16(1):430. doi: 10.1186/s12864-015-1628-8 (PMC4455280; doi:10.1186/s12864-015-1628-8)
Supplement: Additional file 3: Table S1. — Average accuracies of prediction of cross-validated explained genotypic variance for BayesCπ and RR-BLUP for resistance against Fusarium head blight (FHB) and Septoria tritici blotch (STB) based on genotyping data from a 90 k SNP array. [file 12864_2015_1628_MOESM3_ESM.docx]

**Additional file 3: Table S1**

Average accuracies of prediction of cross-validated explained genotypic variance for BayesCπ and RR-BLUP for resistance against Fusarium head blight (FHB) and Septoria tritici blotch (STB) based on genotyping data from a 90 k SNP array.

| Method |  | FHB |  | STB |
| --- | --- | --- | --- | --- |
| **BayesCπ** |  |  |  |  |
| T0 |  |  |  |  |
| Additive & Dominance |  | 0.576 |  | 0.233 |
| Additive |  | 0.550 |  | 0.206 |
| Dominance |  | 0.212 |  | 0.123 |
| T1 |  |  |  |  |
| Additive & Dominance |  | 0.791 |  | 0.639 |
| Additive |  | 0.768 |  | 0.608 |
| Dominance |  | 0.250 |  | 0.240 |
| T2 |  |  |  |  |
| Additive & Dominance |  | 0.958 |  | 0.917 |
| Additive |  | 0.929 |  | 0.890 |
| Dominance |  | 0.291 |  | 0.298 |
|  |  |  |  |  |
| **RR-BLUP** |  |  |  |  |
| T0 |  |  |  |  |
| Additive & Dominance |  | 0.592 |  | 0.255 |
| Additive |  | 0.571 |  | 0.252 |
| Dominance |  | 0.277 |  | 0.088 |
| T1 |  |  |  |  |
| Additive & Dominance |  | 0.798 |  | 0.643 |
| Additive |  | 0.782 |  | 0.638 |
| Dominance |  | 0.358 |  | 0.220 |
| T2 |  |  |  |  |
| Additive & Dominance |  | 0.958 |  | 0.911 |
| Additive |  | 0.943 |  | 0.906 |
| Dominance |  | 0.428 |  | 0.307 |
